# Uptake and Evaluation of a Crosslinguistic Nonword Repetition Test in Clinical and Research Contexts

**DOI:** 10.1111/1460-6984.70306

**Published:** 2026-08-13

**Authors:** Kamila Polišenská, Shula Chiat

**Affiliations:** ^1^ Department of Allied Health City St George's, University of London London UK

**Keywords:** DLD, language assessment, multilingualism, nonword repetition, survey

## Abstract

**Introduction:**

The Crosslinguistic Nonword Repetition Test (CL‐NWR) was designed to accommodate the phonological diversity of human languages, providing a tool that may support the identification of Developmental Language Disorder (DLD) across different linguistic backgrounds. The test materials, including a PowerPoint game with recorded stimuli, administration guidelines, and scoring instructions, were available upon request. This study examines the uptake and application of the CL‐NWR in clinical and research settings through a survey of individuals who have accessed the materials. The survey covered three main areas: (i) participant demographics; (ii) experience of using the CL‐NWR task; and (iii) views on administration and scoring of the CL‐NWR task.

**Methods:**

We explored views of CL‐NWR users through an online survey delivered through the Qualtrics platform and distributed to 156 individuals who had requested CL‐NWR materials up to November 2024. Follow‐up reminders were sent at one‐ and two‐week intervals to maximise response rates. Response formats included yes/no, multiple choice, Likert scales and open‐ended text responses. Survey data were analysed through descriptive statistics and content analysis of open‐ended questions.

**Results:**

A total of 109 respondents from 36 countries completed the survey, yielding a response rate of 70%. The test was most commonly used with multilingual children and those with suspected DLD. Notably, 92% of respondents indicated that the CL‐NWR made a valuable contribution to their assessments. Qualitative responses highlighted its practical benefits as well as areas for refinement.

**Conclusion:**

The high response rate and overwhelmingly positive feedback suggest a high level of engagement and interest in the CL‐NWR, particularly in multilingual populations. These findings will inform future developments of the CL‐NWR test, ensuring its continued relevance and effectiveness in both clinical and research applications. The study also underscores the value of crosslinguistic tools in supporting equitable language assessment worldwide.

**WHAT THIS PAPER ADDS:**

*What is already known on this subject*

- Nonword repetition tasks are widely used to support the identification of Developmental Language Disorder across languages. The Crosslinguistic Nonword Repetition Test (CL‐NWR) was developed as a language‐neutral tool for multilingual populations, and a growing body of research has reported findings from diverse samples using the task. However, its uptake, usability, and application in clinical and research settings have not yet been systematically evaluated.

*What this study adds to existing knowledge*

- This study provides the first large‐scale evidence of global uptake and application of the CL‐NWR across clinical and research contexts. Findings show high levels of use, particularly with multilingual children, and strong perceived value in assessment. The results highlight both the practical utility of the tool and areas for further development.

*What are the clinical implications of this study?*

- The CL‐NWR is a feasible and valuable tool for supporting language assessment, particularly in linguistically diverse populations. The identified need for clearer guidance on administration, scoring, and developmental norms has informed the provision of additional materials and access instructions, supporting more consistent and informed use in clinical and research practice.

## 1 Introduction

Language assessments that are used to identify language disorder in children are generally created for a specific language and standardised on a monolingual population. If children have reduced exposure to the test language, for example if a different language is spoken at home, low performance on the test could be due to limited experience of the language rather than language disorder. Furthermore, there are many languages for which assessments have not been created. These issues pose significant challenges for clinicians assessing multilingual children and children speaking languages for which no assessments are available. The Crosslinguistic Nonword Repetition test (CL‐NWR) was created to address these issues (Chiat 2015).

Nonword repetition has attracted widespread research interest since the first reports that children with language disorder have difficulty repeating made‐up words and the proposal that difficulty repeating nonwords may be an indicator of language disorder (Conti‐Ramsden et al. 2001, Dollaghan and Campbell 1998). This interest has led to the creation of nonword repetition tests with varied characteristics and in many languages. Since nonwords are new to all children whatever their language experience and knowledge, nonword repetition held potential for unbiased assessment of children with limited exposure to the language of assessment. Two recent systematic reviews have examined the diagnostic accuracy of nonword repetition (NWR), one focusing on bilingual children (Ortiz 2021), the other including both bilingual and monolingual populations (Schwob et al. 2021). Schwob et al. (2021) found that the overall mean effect size across studies was large, with children with Developmental Language Disorder (DLD) performing below their typically developing (TD) peers. Ortiz reported variation between studies of bilingual children and noted that discrimination was poorer in studies that used more language‐specific tasks.

Most nonword repetition tests are language‐specific, i.e. created for a specific language, with nonwords sharing characteristics of words in that language which may be quite different from characteristics of words in other languages. Where nonwords share phonological characteristics with words children know, children's performance can benefit from knowledge gained through exposure to the test language. Indeed, there is extensive evidence that this is the case: accuracy of nonword repetition is higher for nonwords that are more typical of real words in the language, for example, nonwords that have higher phonotactic probability (Fu, Chan et al. 2024; Fu, Chen et al. 2024; Szewczyk et al. 2018). Conversely, children with reduced experience of the language would have less opportunity to benefit from real word knowledge, and this might put them at a disadvantage on language‐specific tests. This was the rationale for creating the CL‐NWR, with nonwords designed to be maximally compatible with all languages, by avoiding characteristics of words that occur in some languages but not others (see Chiat 2015; Chiat and Polišenská 2016; Chiat and Polišenská, forthcoming for details). The aim was to provide a test that would not be affected by children's experience of any particular language and would help to identify those with language disorder.

A recent study by Polišenská et al. (2025) evaluated a large body of data collected via the CL‐NWR across 15 countries and 18 research teams. Results showed that multilingual children were not disadvantaged on the CL‐NWR compared to their monolingual peers, and socioeconomic status, gender and amount of exposure to the language of testing did not affect children's performance. Crucially, performance was stronger in TD children than children with DLD and children with language concerns, though the difference in the latter group was greater in younger than older children. Thus, the evidence suggests that the CL‐NWR is bias‐free while having clinical potential to distinguish children with typical/low‐risk language development vs children at‐risk of DLD (see also Fu, Chan et al. 2024; Fu, Chen et al. 2024; Boerma et al. 2015; Boerma and Blom, 2021; Hamdani et al. 2025, though Öberg and Bohnacker (2022, 2024) found only a minority of children with language impairment were correctly identified).

The purpose of the present study is to report on feedback on the use of the CL‐NWR in clinical practice and research through a survey of those who had requested it. We explored views of CL‐NWR users through an online survey delivered via the Qualtrics platform. The survey addressed the demographics of those who had requested the CL‐NWR; whether and in what context it has been used; users’ experience of carrying out the test; and their views of its contribution, limitations, and potential enhancement as a clinical resource. This paper reports both the quantitative and qualitative findings.

## 2 Method

A survey methodology was employed to investigate the use of the CL‐NWR task. To ensure transparent and rigorous reporting, the survey findings are presented in accordance with the Checklist for Reporting Results of Internet E‐Surveys (CHERRIES; Eysenbach 2004), provided in Appendix B. The study received ethical approval from City, St George's, University of London, Language and Communication Science Proportionate Review Research Ethics Committee.

### 2.1 Participants and Procedure

The invitation to participate in the CL‐NWR survey was sent to the email addresses of all individuals who had contacted the investigators to request the CL‐NWR materials up to November 2024. Participation was voluntary and anonymous. The email introduced the survey and provided a link to the online survey presented on Qualtrics.

The survey explained the purpose of the research, asked for the respondent's consent to participate, and only those who gave consent proceeded to the questions. The questions covered demographic characteristics and professional experience of the respondent; whether and how frequently they had administered the CL‐NWR; to whom they had administered it (in terms of clinical or research groups); their purpose in administering it; what it contributed and how useful they found it; and suggestions and recommendations for enhancing its potential as a clinical and research tool.

The survey was designed to take about seven minutes to complete (unless the respondent chose to comment more extensively) and participants were informed about this before they gave consent. Mail merge was used to personalise the email invitations. Following recommendations for enhancing survey response rates (Menon and Muraleedharan 2020), the initial email invitation was followed by a second invitation one week later and a final reminder two weeks after the original email. These were sent to all potential participants because the questionnaire was anonymous, so we did not know who had already responded. Out of the 171 emails sent, 13 were returned as undelivered, and three had an automatic response, one of which provided a new email address to which the link was re‐sent, resulting in a total of 156 invitations successfully delivered. IP addresses were collected by Qualtrics solely for the purpose of identifying and eliminating duplicate submissions. The survey was run between 18 November 2024 and 16 December 2024. Survey completion times were positively skewed; therefore, median completion time is reported. The median completion time was 4.30 min (IQR = 2.85–7.82 min).

### 2.2 Data Analyses

Descriptive data are presented to provide insight into the uptake and use of the CL‐NWR. The total number of responses varied across questions, as the survey used an algorithm to guide participants based on their previous answers. For example, respondents who indicated they had never used the CL‐NWR were asked about their reasons for not using it, while those who had used it were asked follow‐up questions about how they used it and its perceived usefulness. Accordingly, each question is reported separately, with proportions and absolute numbers calculated based on the number of respondents who reached that specific item. Open‐ended comments were analysed using a content analysis. Initial codes were developed inductively from the full dataset and organised into a structured coding framework. After coding was completed, a subset of responses was independently coded by a second rater to assess inter‐rater reliability and ensure consistency in code application. Finally, related codes were grouped into broader categories to summarise common experiences and perceptions regarding the use of the CL‐NWR.

## 3 Results

### 3.1 Participants and Demographics

The invitation emails were delivered to 156 individuals, 111 of whom submitted the questionnaire. Duplicate database entries having the same IP number were eliminated before analysis. Only two IP addresses were identified as identical, leading to the exclusion of one entry; the first entry was kept for data analysis, leading to a sample of *n* = 110. Surveys which only included the consent form but no further data were also eliminated from the database. This resulted in removal of one entry, leaving *n* = 109 entries for analyses.

With 109 valid individual responses to the survey, the response rate was 70%. Participants came from 36 different countries, presented in order of proportion in Table 1.

**TABLE 1:** Participant distribution by country (*n* = 109).

Country	*n*	%
United Kingdom of Great Britain and Northern Ireland	16	15%
Germany	13	12%
United States of America	11	10%
Canada	9	8%
Switzerland	8	7%
Denmark	4	4%
Netherlands	4	4%
Hong Kong	3	3%
Malta	3	3%
Sweden	3	3%
Other countries	35	32%

*Note*: Percentages are rounded to the nearest whole number. *Other countries* represent those with low frequencies (*n* = 1 or *n* = 2 per country) and include Argentina, Australia, Austria, Belgium, Brazil, Croatia, Finland, France, Ireland, Israel, Italy, Japan, Lebanon, Lithuania, Malaysia, New Zealand, Norway, Pakistan, Philippines, Portugal, Singapore, Slovakia, South Korea, Spain, Turkey, and Vietnam.

Table 2 provides a breakdown of respondents’ professional backgrounds, and Table 3 documents their professional and clinical experience in terms of years of working clinically and/or in research. The majority were researchers/academics and/or speech and language therapists/pathologists (SLT/SLPs), accounting for 91% of respondents. While researchers/academics were the largest group, around one third of these also identified as SLT/SLPs, and a further 28% of the respondents were only SLT/SLPs. Most participants had more than 10 years of experience working as an SLT/SLP (59%) or as a researcher (52%). Respondents were able to select more than one professional role, so individuals who identified as both clinicians and researchers were included in both categories and responded to all relevant follow‐up questions.

**TABLE 2:** Overview of professional backgrounds of respondents (*n* = 109).

Primary Role	Total (*n*)	Percentage
Researcher/Academic	46	42%
SLT/SLP	31	28%
SLT/SLP + Researcher/Academic	22	20%
Educational Professional	2	2%
Educational Professional + Researcher/Academic	4	4%
Other combinations	4	4%

*Note*: Other combinations included: SLT/SLP + Teacher, SLT/SLP + Academic + Teacher, SLT/SLP + Other, Not further specified.

**TABLE 3:** Overview of participants’ clinical and/or research experience.

	SLT/SLP role (*n* = 56)	%	Researcher role (*n* = 73)	Percentage
Less than 1 year	1	2%	2	3%
1–3 years	7	13%	11	15%
4–7 years	8	14%	15	20%
8–10 years	7	13%	7	10%
More than 10 years	33	59%	38	52%

In terms of highest qualification, approximately half of the sample held a doctoral degree (PhD; 55/109), 36% had a Master's degree (40/109), 10% had a Bachelor's Degree (11/109) and 3% other (3/109).

The majority of respondents reported that their clients or participants typically fell into the primary school (6–12 years; 83%, 90/109) and preschool (3–5 years; 75%, 82/109) age ranges. Smaller proportions worked with infants (0–2 years; 17%, 18/109), adolescents (13–18 years; 16%, 17/109), and adults (19+ years,11%, 12/109).

### 3.2 Uptake of the CL‐NWR

Almost three‐quarters of respondents (72%, 78/109) had used the CL‐NWR since receiving it, with the remaining 28% (31/109) reporting that they had not administered it. Uptake of the task was spread across 32 out of the 36 countries where respondents requested it. The majority of those who used it administered it more than 10 times (49%, 38/78), followed by 1–3 times (34%, 27/78) and 4–10 times (17%, 13/78). Table 4 breaks down the usage by professional role and shows that researchers and SLT/SLPs showed similar patterns of usage, with most using the task more than 10 times and about a quarter never using it. The proportion of never using the task was higher in the role of teacher and other, however, those roles were infrequent amongst the respondents (refer to Table 2).

**TABLE 4:** Breakdown of CL‐NWR usage frequency by professional role (Percentages and Counts).

Role/Usage	Never	1–3 times	4–10 times	10 times+	Total
SLT/SLP	23% (13)	32% (18)	13% (7)	32% (18)	100% (56)
Researcher	23% (17)	18% (13)	12% (9)	47% (34)	100% (73)
Teacher	37% (3)	25% (2)	13% (1)	25% (2)	100% (8)
Other	50% (1)	50% (1)	0% (0)	0% (0)	100% (2)

Given that the CL‐NWR was developed to support clinical assessment, we were particularly interested in its use by clinicians who were not also researchers. Of the 33 clinicians, 11 never used the task (33%), 14 used it 1–3 times (43%), four used it 4–10 times (12%) and a further four used it more than 10 times (12%). In summary, the majority of clinicians had used the CL‐NWR, with many reporting at least occasional use of the task.

### 3.3 Administration and Scoring

The majority of respondents (97%, 75/77) reported that they were confident in administration and scoring of the CL‐NWR. Similarly, most were confident in their ability to interpret results (91%, 70/77) (see Table 5).

**TABLE 5:** Level of confidence in administration, scoring and interpretation of results.

	Administration and scoring		Ability to interpret results	
	*n*	%	*n*	%
Very confident	43	56%	30	39%
Somewhat confident	32	41%	40	52%
Not confident	2	3%	7	9%

**FIGURE 1:**
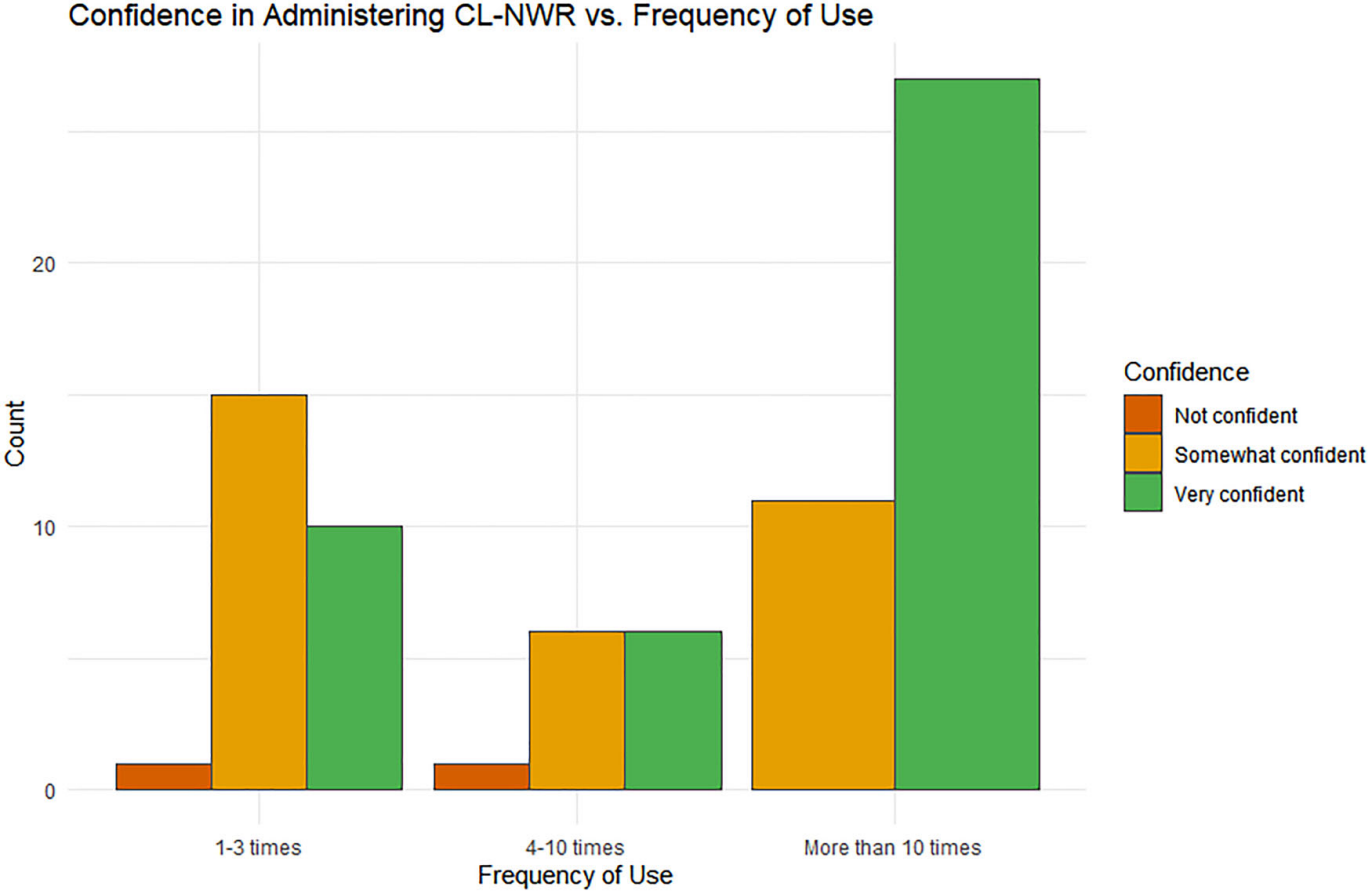
Relationship between frequency of use of the CL‐NWR and level of confidence.

The ease of administration and scoring was also rated highly, with 73% (56/77) selecting *very easy* and 27% (21/77) *neither easy nor difficult*. Time‐efficiency of the CL‐NWR also scored highly, with 70% of respondents (54/77) seeing the task as *very efficient*, 29% (22/77) as *somewhat efficient* and only 1% (1/77) as *not efficient*. Examining the confidence in administering the CL‐NWR in relation to frequency of use (see Figure 1), there appeared to be a relationship such that respondents selecting *very confident* also had experience of administering the task more than 10 times.

### 3.4 Use of the CL‐NWR

Use of the CL‐NWR was most frequent with bilingual/multilingual children (77%) and children suspected to have DLD (54%), with smaller proportions using it for other groups (see Table 6). The survey question relating to use of the CL‐NWR did not initially display for all eligible participants. This omission was identified while the survey was live and subsequently corrected. As a result, the number of respondents for this item is lower than for other questions (*n* = 26), see Table 6.

**TABLE 6:** Usage of tasks across groups (with participants asked to select all that apply, *n* = 26).

Group	Percentage of respondents	*n*
Bilingual/multilingual children	77%	20
Children suspected to have DLD	54%	14
Research participants	35%	9
Children referred for clinical assessment	27%	7
Children diagnosed with DLD or language difficulties	23%	6
Monolingual children	23%	6
Children identified by nursery/school professionals	8%	2
Children with speech difficulties	8%	2
Other purposes	8%	2

The majority of respondents reported that in their experience, CL‐NWR results aligned with other diagnostic assessments: 9% (6/70) selected *always* aligned and 57% (40/70) selected *often* aligned; 31% (22/70) selected *sometimes*, and only 3% (2/70) reported that they aligned *rarely*.

Turning to questions about their view of the task, 92% of the respondents who used the task and answered this question (69/75) selected *yes* when asked whether the *CL‐NWR made a useful contribution to their assessment;* with 8% (6/75) selecting *no*. The most common reason selected was contribution to a research study (68%, 47/69). This was followed by: indicating strengths or limitations in phonological processing and memory (58%, 40/69), indicating risk of DLD (54%, 37/69), guiding further assessment (22%, 15/69), helping to discuss the child's skills with parents or other professionals (20%, 14/69), and other reasons (3%, 2/69). Finally, 76% of respondents (79/105) agreed with the statement that *the CL‐NWR is a valuable tool for advancing research on DLD and bilingualism*, 22% (23/105) selected *maybe* and only 2% (2/105) did not think it was a valuable tool.

### 3.5 Reasons for Not Using the Task

Respondents who reported that they did not administer the task (28%, 31/109) were asked *what the reason was for not administering the CL‐NWR*. They could select more than one option. The most common reason was *Other* (55%, 17/31). This was followed by *the tool not being suitable for the respondent's clients or research participants* (31%, 10/31), followed by *lack of norms* (16%, 5/31). Equal proportions selected not using it due to *uncertainty about what it measures* (13%, 4/31), or *uncertainty about scoring* (13%, 4/31), while 10% (3/31) were unsure about *administration*. For respondents who indicated that the CL‐NWR did not make a useful contribution to their assessment (6/75), a follow‐up question was asked about *why the CL‐NWR was not useful*. Respondents were invited to select all the options that applied. The most common reason why it was not useful was being unsure what it told them (67%, 4/6). This was followed by difficulty in administering or scoring (33%, 2/6), and both lack of norms and other reasons (each 17%, 1/6).

### 3.6 Suggestions and Recommendations

Looking to future support for use of the CL‐NWR test, the most common request was for access to case studies or examples, cited by 62 respondents. This was followed by scoring examples or templates, mentioned by 57 respondents, and online training or video demonstrations, selected by 40 respondents. More detailed administration guidelines were requested by 30 respondents, while 13 respondents suggested other forms of support.

### 3.7 Qualitative Analysis

The survey included two open‐ended questions:

- *Have you used the CL‐NWR in a research study? If yes, please describe how it contributed to your research findings*.
- *If you have any further suggestions or recommendations regarding the CL‐NWR, please share them here*.

Three‐fifths of the participants who submitted the survey (61%, 67/109) entered responses to these questions, spanning 25 countries, of whom 18 identified as practitioner only (clinical or educational) and 49 as researcher or researcher + practitioner.

The two authors independently reviewed all comments to develop an initial set of codes, which were subsequently discussed and refined into a final coding framework. All comments were then coded by the second author, while the first author independently coded a subset of 28 comments to assess reliability. The two authors assigned identical codes to 21 of the 28 comments (75%), indicating substantial agreement according to Landis and Koch (1977).

Table 7 presents the final coding framework and the distribution of coded comments, together with their grouping into broader categories. See Appendix A for the definition of these codes.

**TABLE 7:** Coding framework for qualitative data analysis with a rate of occurrence.

Codes	Broader categories
Positive engagement (*n* = 8)	Views on the assessment (*n* = 14)
No bias (*n* = 6)	
Screening (*n* = 3)	Positive contribution to assessment (*n* = 35)
Diagnosis/difficulties/abilities (*n* = 22)	
Complementing other measures (*n* = 10)	
Norms (*n* = 13)	Limitations and suggestions (*n* = 27)
Design and clarity (*n* = 14)	
Project in progress: Reported use (*n* = 8)	Limited use and non‐use (*n* = 21)
Non‐use (*n* = 13)	
Other (*n* = 6)	Unclassified (*n* = 6)
Total (*n* = 103)	Total (*n* = 103)

#### 3.7.1 Views on the Assessment

Many respondents reported positive experiences with the format, content, and overall use of the CL‐NWR. The task was frequently described as engaging for children and straightforward to administer, contributing to its perceived value in both clinical and research contexts.

Participants consistently highlighted children's enjoyment of the task, particularly the game‐like “necklace” format. For example, one respondent (ID027, SLT/SLP, Researcher) noted that “*(…) it is by far the test children enjoy the most. Some even want to do it all over again once we finish*.” Similarly, another respondent (ID051, Researcher) described being *“(…) pleasantly surprised by the children's enjoyment of the necklace task and the ease of administration. (…)*” The mixed item lengths, initially expected to increase difficulty, were instead reported to enhance engagement for both children and examiners: *“(…) it seems to be more enjoyable for the examiners and children during administration*. (…)” In addition to clinical and research use, the CL‐NWR was reported to have value in teaching contexts. One respondent commented that “*the task and writings about the quasi‐universal NWR task have been very useful for teaching purposes also*.”

Respondents also highlighted the test's lack of bias, making it suitable for use with multilingual children. In particular, participants noted its usefulness in contexts where standardised assessments are unavailable for specific heritage languages. For example, one respondent (ID067, Researcher) reported that *“(…) it could be used for multiple heritage languages for which other assessments were not available*.”

The CL‐NWR was also described (ID030, Researcher) as supporting more equitable identification of DLD in multilingual populations, as it is “*not biased against children with less experience with a particular language*.” Relatedly, researchers indicated that the task could assist in decision‐making regarding group classification, for example helping to determine whether multilingual children should be assigned to DLD or TD groups.

Two respondents expressed negative views regarding the CL‐NWR and its contribution to assessment. Reported issues related to practical aspects of administration and uncertainty about the usefulness of the results. For example, one respondent (ID087, SLT/SLP) suggested a different format for the assessment might work better: *“(…) The powerpoint doesn't always work, I have issues with using it on the iPad which is what I primarily use for assessments. The sound quality of the recordings could be better (…)”*. Another respondent expressed uncertainty regarding interpretation, commenting: “*Not sure I will use the results*.”

#### 3.7.2 Positive Contribution to Assessment

Approximately one third of respondents reported that the CL‐NWR contributed meaningfully to language assessment. Most commonly, this related to the identification of DLD and/or the nature of language abilities and difficulties. A small number of respondents explicitly referred to the use of the CL‐NWR as a screening tool. For example, one participant (ID088, SLT/SLP, Educational professional, Researcher) reported: “*I used it as a screener for language skills among children aged 5–6 year old. It displayed a uniformity in performance among the cohort”*, while another (ID066, Researcher) described it as *“(…) a promising tool for primary assessment and potentially an accurate screener to distinguish children with DLD from their TD peers for extra language assessments”*.

Many respondents highlighted the role of the CL‐NWR in identifying language difficulties and distinguishing between children with and without DLD. For instance, one respondent (ID013, SLT/SLP) stated that “*It helped to differentiate those with and without DLD,”* while another (ID063, Researcher) reported using the task to assess underlying skills: *“we used it to measure the participants' short term/ working memory”*.

The CL‐NWR was also frequently described as complementing other assessment tools within a broader battery. Respondents (ID097, Researcher) noted that “*It was a marker for DLD among others (ex. Sentence repetition and narrative discourse)”*, and (ID068, Researcher) *“(…) it contributed to the overall evaluation of participants language performance (…)”*. In some cases, the task was also used alongside measures of executive skills (ID061, Researcher: “*This complemented the tests I did on inhibition in preschool monolingual and bilingual children assessed as being at risk of developing a language disorder*)”, supporting a more comprehensive understanding of children's profiles (ID043, Researcher: *“(…) the task revealed patterns that highlighted the role of phonological deficits in reading and language difficulties, contributing significantly to the understanding of dyslexia and related learning challenges”*.

#### 3.7.3 Limitations and Suggestions

Respondents also identified several limitations of the CL‐NWR and provided suggestions for future development. Unsurprisingly, and in line with responses to closed questions, the most common issue was the need for norms and age‐related cut‐offs. Respondents emphasised that the availability of norms would substantially enhance the clinical utility of the task. For example, one participant (ID049, SLT/SLP) stated: “*Interested in norms!!”*, while another (ID084, SLT/SLP) commented “*I really like the assessment but it would be much more valuable with norm/criterion scores”*.

A notable number of comments were on details of materials, raising issues with stimuli, or querying design of stimuli. One respondent (ID062, Researcher) shared concerns about the suitability of some stimuli across different languages: “*some item choices are problematic for some language combinations (…)”*. Others (ID011, SLT/SLP, Researcher) questioned whether the simplicity of the stimuli might reduce sensitivity for older samples: *“(…) The design of the CL‐NWR and the thought you put into it seem excellent. I worry, however, that the simplicity of the word structures necessitated by going as universal as possible will render the test less sensitive to DLD, especially in older children and adults. (…)”*.

Respondents also put forward suggestions for improving scoring procedures, for example (ID086, SLT/SLP, Researcher): “*When scoring, it would be helpful to already have the numbers allotted on the page/form separated by syllabic length (…)”*. A smaller number called for further guidance on the use of the CL‐NWR, for example, a question (ID034, SLT/SLP) was raised regarding the amount of language exposure a child would need before being administered the task: “*Would you recommend that the CL‐NWR be used for children just starting their exposition in the language of schooling? Or you would recommend a minimum of exposition in the language of testing (ex. 12 months)”*. Others (ID060, SLT/SLP) called for support with interpreting the results: *“Could you give me information on how to interpret the results of the assessment and what further assessment would be indicated”*.

### 3.8 Limited Use and Non‐use

Just under a quarter of comments referred either to non‐use of the CL‐NWR or to ongoing projects that had not yet yielded views on its contribution. All reasons for non‐use were practical such as changes of job, lack of grant funding, problems recruiting participants.

## 4 Discussion

The survey addressed three main areas related to the CL‐NWR uptake and use: (i) participant demographics; (ii) experience of using CL‐NWR; and (iii) views on administration and scoring.

### 4.1 Participant Demographics

Participants were drawn from 36 countries, with the largest proportions from the United Kingdom (15%), Germany (12%), and the United States (10%). The remaining respondents were distributed across a wide range of countries, each contributing smaller proportions. This broad geographical spread highlights the international reach of the CL‐NWR and its uptake across diverse linguistic, academic and clinical contexts.

The sample was predominantly composed of researchers/academics (42%), followed by speech and language therapists (SLTs/SLPs; 28%), with a further 20% reporting combined roles in research and clinical practice. Only a small proportion of respondents identified as educational professionals or other role combinations. This distribution suggests that uptake of the CL‐NWR has so far been strongest amongst researchers (clinical or non‐clinical). This is unsurprising since the CL‐NWR was developed as part of a research project and at the time of the survey could only reach practitioners through dissemination by researchers and/or by discovering it through research papers that included information about how to obtain the materials.

The sample was highly educated, with approximately half of respondents holding a doctoral degree and over one third a Master's degree. This is not unexpected given the predominance of researchers in the sample, as well as the fact that a Bachelor's degree is typically required for entry into the profession, and in many countries a Master's degree is necessary to qualify as an SLT/SLP.

### 4.2 Experience of Using CL‐NWR

Responses to closed questions were overwhelmingly positive about the CL‐NWR's contribution. While the most frequently cited contribution was to research studies, a majority gave reasons related to the assessment process, valuing it as an indicator of risk of DLD or phonological processing and memory skills. About a fifth of respondents found it helpful post‐assessment, guiding further assessment or discussion of a child's skills with others involved. Open‐ended questions aligned with and elaborated these selections. In line with the rationale for and aims of the test, respondents highlighted its contribution to identification of difficulties, usually as part of a battery or alongside other assessments, and of particular value with multilingual children given its non‐biased design. These comments are in line with the reasoning behind the creation of the CL‐NWR as a task which minimises the potential contribution of language knowledge while drawing on skills that underpin language development and may be impaired in children with DLD. At the same time, they highlight the importance of combining the CL‐NWR with tasks drawing on other skills that underpin typical and atypical language development.

In addition, several respondents highlighted the child‐friendly nature of the task. The game‐like necklace activity was reported to be engaging and enjoyable for children, which may support motivation and attention during assessment. This is an important consideration in both clinical and research contexts as positive engagement can facilitate more accurate assessment and reduce the likelihood of non‐compliance. The reported acceptability of the CL‐NWR therefore represents a notable strength, particularly when working with younger populations or those who may be less responsive or familiar with more traditional assessment formats.

A number of participants queried aspects of the stimuli that make the test maximally compatible with diverse lexical typologies, the key aim of the test. The very limited internal structure, with simple CV syllables and a limited range of consonants and vowels, was necessary to be compatible with languages that lack clusters and with the very wide variation in consonant and vowel inventories. This makes items less challenging, potentially reducing differences between typically and non‐TD children. The ‘robotic’ prosody of items was chosen to be as neutral as possible between the wide crosslinguistic variation in lexical prosody (Chiat 2015). This makes items artificial‐sounding, potentially reducing their relevance to real word processing. Recognising the rationale for these characteristics, evidence from rigorous research studies and clinical experience are vital to determine whether the test makes a useful contribution despite its peculiar characteristics. For this reason, the positive views on the format and contribution of the test are encouraging, as is the recognition that it should be complemented with other assessments.

Our invitation to participate explicitly asked people to respond to the survey even if they had not used the task, and the survey questions gave opportunities for negative feedback. Just under 30% of respondents had not used the task, the majority for ‘Other reasons’. Only small proportions selected lack of norms or uncertainties about the test, and these same reasons were selected by the small number of respondents who had administered the test but did not find it useful. Again, the quantitative data were matched by responses to the open‐ended questions. Some participants who had not administered the test took the opportunity to explain the practical reasons for this, and although a notable proportion of comments highlighted the need for norms, or requested clarification of methods or interpretation, these limitations were not in general a barrier to using the test and reporting it to be useful. Only two comments were directly negative.

### 4.3 Administration and Scoring of CL‐NWR

Some of the specific issues raised have already been addressed by amendments to the instructions for scoring made since the test was first available, providing clearer specification of allowances for variants in children's repetition of target consonants and vowels. Others will be considered for further refinement of these instructions. While no respondent commented on whole‐item scoring as being particularly challenging, this could be an issue. With just 16 items, uncertainty about one or two items can make a difference in total score that affects decision‐making based on cut‐offs. Whole‐item scoring was originally applied as research had found it to be as discriminating as phoneme scoring (see review in Schwob et al. 2021), and it is quicker and therefore more clinically efficient. Scoring at syllable level has been applied in a study of Cantonese‐speaking children and found to be informative (Fu et al. 2024, and see Chiat and Polišenská, forthcoming). Scoring accuracy of syllables rather than whole items is also quick, and it may be less challenging than whole‐item scoring since uncertainty about one or two syllables will have less impact on overall score and clinical decision‐making. The need for norms and age‐related cut‐offs, widely expressed in responses to both closed and open‐ended questions, is obviously central for application in clinical practice, supporting decision‐making on children's performance and next steps. While norms are not yet available for individual languages or crosslinguistically, a recent analysis of secondary data (Polišenská et al. 2025) collected by 18 teams in 15 countries now provides age‐related cut‐offs as an indicator of risk for language disorder. Further research should support the establishment of norms and help determine the extent to which universal norms and clinical cut‐offs can be established. This will indicate whether norms and cut‐offs are valid for children from different language backgrounds, including those with minimal exposure to the language of testing, and will help establish the lower and upper age limits for which the CL‐NWR is informative.

Looking at confidence in administering the CL‐NWR and frequency of use (see Figure 1), there appears to be a positive relationship, with respondents reporting high confidence also tending to report more frequent use (e.g., more than 10 administrations). This pattern may reflect increased confidence through repeated exposure and practice, although it could also be that people who are confident are more likely to administer the task more often. In this context, the reported need for additional guidance on administration, scoring and interpretation is particularly noteworthy. This need, identified in both qualitative responses and closed‐question data, suggests that confidence and frequency of use may be further supported through targeted resources. In particular, respondents frequently selected case studies, scoring examples, and online training or videos as desirable support. Such resources may be critical in facilitating the transition of the CL‐NWR from an evidence‐based research tool to a more widely adopted and consistently implemented clinical assessment.

### 4.4 Limitations

While the study provides valuable insights, several limitations should be acknowledged. The sample was composed predominantly of highly qualified professionals, including those engaged in research and advanced clinical practice. As a result, the findings may not fully reflect the perspectives and experiences of less experienced practitioners, potentially limiting the generalisability of the results regarding the uptake and evaluation of the CL‐NWR. A further limitation is that the sample consisted of individuals who had previously requested access to the CL‐NWR, and therefore does not represent an open or unbiased sample. It is possible that those who requested the test before it was publicly available and who chose to respond to this survey constitute a biased group, as they are likely to welcome a crosslinguistic assessment and be enthusiastic about research evidence of its clinical potential. Furthermore, although the response rate was high, it is possible that individuals who chose to respond differ systematically from those who did not. Due to the anonymous nature of the survey, it was not possible to examine the characteristics of non‐responders. Future research should replicate the survey with larger and even more diverse samples, including clinicians working in school and community settings who are not directly involved in research in order to capture a broader range of perspectives on the use of the CL‐NWR. While responses to the survey highlighted the CL‐NWR's ease of administration, child‐friendliness, and contribution to screening and assessment alongside other assessments, its ultimate purpose is to support clinical practice with multilingual children. The extent to which these findings generalise beyond the current sample calls for further investigation.

## 5 Conclusions

This study provides the first systematic evidence of the global uptake and application of the CL‐NWR across clinical and research contexts. The findings demonstrate strong engagement with the task, particularly in multilingual populations, and highlight its value as part of a broader assessment battery. Respondents emphasised its ease of administration, child‐friendly design, and potential to support equitable language assessment. At the same time, the results underline the importance of continued developments to support clinical implementation, particularly through the provision of guidance on administration, scoring, and interpretation, as well as the development of normative data. The CL‐NWR and its accompanying materials and guidance are now freely available to support uptake in both research and clinical settings. It can be accessed through a Qualtrics link provided in the Informed SLP's report of the CL‐NWR, and repeated here.

Overall, the findings of this study support the CL‐NWR as a promising tool for multilingual language assessment and emphasise the need for assessment approaches that reduce linguistic bias in the identification of DLD. Responses in this survey further suggest broad support for the CL‐NWR's potential, with a large majority of respondents indicating that the CL‐NWR is a valuable tool for advancing research on DLD and multilingualism.

## Conflicts of Interest

The authors declare no financial conflicts of interest. The authors were involved in the development of the CL‐NWR evaluated in this study and therefore have a non‐financial interest in the work.

## Supporting information




**Supporting Information**: jlcd70306‐supp‐0001‐SuppMat.docx

## Data Availability

The raw/processed data required to reproduce the above findings cannot be shared at this time due to legal/ethical reasons.

## References

[jlcd70306-bib-0001] Boerma, T. , S. Chiat , P. Leseman , M. Timmermeister , F. Wijnen , and E. Blom . 2015. “A Quasi‐Universal Nonword Repetition Task as a Diagnostic Tool for Bilingual Children Learning Dutch as a Second Language.” Journal of Speech, Language, and Hearing Research 58, no. 6: 1747–1760. 10.1044/2015_JSLHR-L-15-0058. 26444988

[jlcd70306-bib-0002] Boerma, T. D. , and W. B. T. Blom . 2021. “Crosslinguistic Nonword Repetition and Narrative Performance Over Time: A Longitudinal Study on 5‐ to 8‐Year‐old Children With Diverse Language skills.” In Language Impairment in Multilingual Settings: LITMUS in Action Across Europe, edited by S. Armon‐Lotem , and K. Grohmann , (302–328). John Benjamins.

[jlcd70306-bib-0003] Chiat, S. 2015. “Nonword Repetition.” In Assessing Multilingual Children: Disentangling Bilingualism From Language Impairment, edited by S. Armon‐Lotem J. Jong , and N. Meir (125–150). Multilingual Matters.

[jlcd70306-bib-0004] Chiat, S. , and K. Polišenská . 2016. “A Framework for Crosslinguistic Nonword Repetition Tests: Effects of Bilingualism and Socioeconomic Status on Children's Performance.” Journal of Speech, Language, and Hearing Research 59, no. 5: 1179–1189. 10.1044/2016_JSLHR-L-15-02. 27750281

[jlcd70306-bib-0017] Chiat, S. , and K. Polišenská (Forthcoming). “Nonword Repetition.” In Assessing Multilingual Children, edited by S. Armon‐Lotem J. Jong , and N. Meir , 2nd edition. Multilingual Matters.

[jlcd70306-bib-0005] Conti‐Ramsden, G. , N. Botting , and B. Faragher . 2001. “Psycholinguistic Markers for Specific Language Impairment (SLI).” Journal of Child Psychology and Psychiatry 42, no. 6: 741–748. 11583246 10.1111/1469-7610.00770

[jlcd70306-bib-0006] Dollaghan, C. , and T. F. Campbell . 1998. “Nonword Repetition and Child Language Impairment.” Journal of Speech, Language, and Hearing Research 41, no. 5: 1136–1146. 10.1044/jslhr.4105.1136 9771635

[jlcd70306-bib-0007] Eysenbach, G. 2004. “Improving the Quality of Web Surveys: The Checklist for Reporting Results of Internet E‐Surveys (CHERRIES).” Journal of Medical Internet Research 6, no. 3: e34. 15471760 10.2196/jmir.6.3.e34 PMC1550605

[jlcd70306-bib-0008] Fu, N. C. , A. Chan , S. Chen , K. Polišenská , and S. Chiat . 2024. “Revisiting Nonword Repetition as a Clinical Marker of Developmental Language Disorder: Evidence From Monolingual and Bilingual L2 Cantonese.” Brain and Language 257: 105450. 10.1016/j.bandl.2024.105450. 39305719

[jlcd70306-bib-0009] Fu, N. C. , S. Chen , K. Polišenská , A. Chan , R. Kan , and S. Chiat . 2024. “Nonword Repetition in Children With Developmental Language Disorder: Revisiting the Case of Cantonese.” Journal of Speech, Language, and Hearing Research 67, no. 6: 1772–1784. 10.1044/2024_JSLHR-22-00397. 38683057

[jlcd70306-bib-0010] Hamdani, S. , A. Chan , R. Kan , et al. 2025. “Identifying Developmental Language Disorder (DLD) in Multilingual Children: A Case Study Tutorial.” International Journal of Speech‐Language Pathology 27, no. 2: 1–15. 10.1080/17549507.2024.2326095. 38764397

[jlcd70306-bib-0011] Landis, J. R. , and G. G. Koch . 1977. “The Measurement of Observer Agreement for Categorical Data.” Biometrics 33, no. 1: 159–174. 10.2307/2529310. 843571

[jlcd70306-bib-0012] Menon, V. , and A. Muraleedharan . 2020. “Internet‐based Surveys: Relevance, Methodological Considerations and Troubleshooting Strategies.” General Psychiatry 33, no. 5: e100264. 32818170 10.1136/gpsych-2020-100264 PMC7398086

[jlcd70306-bib-0013] Öberg, L. , and U. Bohnacker . 2022. “Non‐Word Repetition and Vocabulary in Arabic‐Swedish‐Speaking 4–7‐Year‐olds With and Without Developmental Language Disorder.” Languages 7, no. 3: 204. 10.3390/languages7030204.

[jlcd70306-bib-0014] Öberg, L. , and U. Bohnacker . 2024. “Beyond Language Scores: How Language Exposure Informs Assessment of Nonword Repetition, Vocabulary and Narrative Macrostructure in Bilingual Turkish/Swedish Children With and Without Developmental Language Disorder.” Children 11, no. 6: 704. 10.3390/children11060704. 38929283 PMC11202042

[jlcd70306-bib-0015] Ortiz, J. A. 2021. “Using Nonword Repetition to Identify Language Impairment in Bilingual Children: A Meta‐Analysis of Diagnostic Accuracy.” American Journal of Speech‐Language Pathology 30, no. 5: 2275–2295. 10.1044/2021_AJSLP-20-00237. 34269597

[jlcd70306-bib-0016] Polišenská, K. , S. Chiat , J. Szewczyk , et al. 2025. “Evaluation of the Crosslinguistic Nonword Repetition Test: Evidence From a Large and Diverse Secondary Data Set.” Journal of Speech, Language, and Hearing Research 68, no. 11: 5363–5383. 10.1044/2025_JSLHR-25-00158. 41044009

[jlcd70306-bib-0018] Schwob, S. , L. Eddé , L. Jacquin , et al. 2021. “Using Nonword Repetition to Identify Developmental Language Disorder in Monolingual and Bilingual Children: A Systematic Review and Meta‐Analysis.” Journal of Speech, Language, and Hearing Research 64, no. 9: 3578–3593. 10.1044/2021_JSLHR-20-00552 34407377

[jlcd70306-bib-0019] Szewczyk, J. M. , M. Marecka , S. Chiat , and Z. Wodniecka . 2018. “Nonword Repetition Depends on the Frequency of Sublexical Representations at Different Grain Sizes: Evidence From a Multi‐Factorial Analysis.” Cognition 179: 23–36. 29902629 10.1016/j.cognition.2018.06.002

