# Supplementary material for: Uptake and Evaluation of a Crosslinguistic Nonword Repetition Test in Clinical and Research Contexts
Source: Int J Lang Commun Disord. 2026 Aug 13;61(5):e70306. doi: 10.1111/1460-6984.70306 (PMC13472312; doi:10.1111/1460-6984.70306)
Supplement: Supplementary file 1 [file JLCD-61-0-s001.docx]

**Appendix A: Codes and their definition**

| **Codes** | **Definition** |
| --- | --- |
| Positive engagement | Reference to the test's value, usefulness, or ease of use |
| No bias | Reference to non-bias and/or explicit reference to benefits for use with multilingual children/populations |
| Screening | Explicit reference to use for screening |
| Diagnosis/difficulties/abilities | Use for identifying DLD or particular language abilities/difficulties |
| Complementing other measures | Benefits of using CL-NWR in combination with other measures |
| Norms | Norms/cut-offs would enhance contribution to clinical assessment |
| Design and clarity | Reference to amendments or recommended changes to materials, methods, interpretation |
| Project in progress: Reported use | Reference to ongoing or planned use |
| Limited use and non-use | Report of and reasons for not using the CL-NWR (having requested it) |
| Other | Other comments not meeting any of the above descriptions |

**Appendix B: Checklist for Reporting Results of Internet E-Surveys (CHERRIES) (Eysenbach, 2004)**

| **Item category** | **Checklist item** | **Y/ N** | **Comments** |
| --- | --- | --- | --- |
| Design | Describe survey design | Y | Target population as described in method section. Purposive sampling. |
| Institutional Review Board (IRB) approval and informed consent process | IRB approval | Y | From City, St George’s, University of London, Language and Communication Science Proportionate Review Research Ethics Committee. |
|  | Informed consent | Y | Participant information was presented in the initial survey distribution email and informed consent was gained via Qualtrics before the survey commenced. |
|  | Data protection | Y | Only the research team has access to password-protected data on Qualtrics. |
| Development and pre-testing | Development and testing | Y | As described in method section. |
| Recruitment process and description of the sample having access to the questionnaire | Open survey vs closed survey | Y | Closed survey |
|  | Contact mode | Y | Initial contact with potential participants was made via email with people who previously requested the CL-NWR task from the research team. |
|  | Advertising the survey | Y |  |
| Survey administration | Web/E-mail | Y | Email |
|  | Context | Y | Individuals who previously made contact and requested the CL-NWR via email form the research team, as described in method section. |
|  | Mandatory/voluntary | Y | Voluntary |
|  | Incentives | Y | Nil financial incentives offered. |
|  | Time/Date | Y | Between 18 November 2024 and 16 December 2024 |
|  | Randomisation of items or questionnaires | N | Nil |
|  | Number of items | Y | Number of items varied depending on answers provided. |
|  | Number of screens (pages) | Y | 10 pages |
|  | Completeness check | Y | Forced-response feature selected on Qualtrics for consent form only. |
|  | Review step | Y | Respondents were allowed to review and change their answers |
| Response rate | Unique site visitor | Y | Collection of IP addresses were enabled to check for duplicate submissions. View rate not applicable as it was not an open survey. |
|  | View rate (Ratio of unique survey visitors/ unique site visitors) | N |  |
|  | Participation rate (Ratio of unique visitors who agreed to participate/ unique first survey page visitors) | N |  |
|  | Completion rate (Ratio of users who finished the survey/users who agreed to participate) | Y | 109/110 x 100% = 99% |
| Preventing multiple entries from the same individuals | Cookies used | N | Cookies were not used to assign unique user identifier given the possibility that some participants may drop out and want to start a survey again. To avoid inclusion of duplicate entries from the same respondents, IP addresses were checked for duplicates. |
|  | IP check | Y | IP addresses were checked for duplicates. Duplicate database entries with the same user ID were excluded before analysis. |
|  | Log file analysis | N |  |
|  | Registration | N | As described above, the IP addresses were checked and where duplicate, first entry was kept for analysis. |
| Analysis | Handling of incomplete questionnaires | Y | Only surveys that terminated before completing the demographic section were excluded. |
|  | Questionnaires submitted with an atypical timestamp | Y | The surveys with time completion of less than 60 seconds were reviewed for completeness. If demographic information was missing, the surveys were excluded. |
|  | Statistical correction | N | No statistical correction was applied, as the sample was purposive rather than representative. |
